# American Society of Anesthesiologists Guidance on Periprocedural Fasting in Patients Receiving Tube Feeding

**DOI:** 10.1097/ao9.0000000000000037

**Published:** 2026-10-28

**Authors:** Jamie L. Sparling, Thomas Edmiston, Prashant Nasa, Ravindra A. Gupta, Kunal Karamchandani, Ashish K. Khanna, Jarva Chow, Federico Bilotta, Alisha Sachdev, Roshni Sreedharan, Shahla Siddiqui, Paul E. Wischmeyer, Christian Stoppe, Jeffrey I. Mechanick, D. Dante Yeh, Ronald G. Pearl, Todd W. Rice, Hassan S. Dashti, Michael DeWane, Martin Rosenthal, Richard P. Dutton, Alexander Nagrebetsky, for the ASA Committee on Critical Care Medicine Task Force on Periprocedural Fasting in Patients Who Receive Tube Feeding, Shahla Siddiqui

**Affiliations:** Department of Anesthesia, Critical Care, and Pain Medicine, Beth Israel Deaconess Medical Center, Harvard Medical SchoolBoston, Massachusetts; Integrated Hospital Care InstituteCleveland Clinic Lerner College of Medicine, Care Western Reserve UniversityCleveland, Ohio; Department of Anesthesiology, University of Minnesota Medical SchoolMinneapolis, Minnesota; Ross University School of MedicineBridgetown, Barbados; Red Bank Anesthesia, Riverview Medical CenterRed Bank, New Jersey; Department of Anesthesiology, Columbia University Irving Medical CenterNew York, New York; Department of Anesthesiology and Pain Medicine, University of Washington Medical Center- MontlakeSeattle, Washington; Department of Anesthesiology, Emory University School of MedicineAtlanta, Georgia; Department of Anesthesiology, University of Vermont Medical CenterLarner College of Medicine, University of VermontBurlington, Vermont; Department of Anesthesiology and Pain Management, University of Texas Southwest Medical CenterDallas, Texas; Department of Anesthesia and Critical Care, University of ChicagoChicago, Illinois; Department of Anesthesiology, Wake Forest University School of MedicineWinston-Salem, North Carolina; Department of Anesthesiology, Wayne State University School of MedicineDetroit, Michigan; Department of Medicine, SUNY Upstate Medical UniversitySyracuse, New York; Department of Anesthesiology and Pain Management, University of Texas Southwestern Medical CenterDallas, Texas; Department of Anesthesiology and Perioperative Medicine, Mayo ClinicRochester, Minnesota; Department of Anesthesiology, Madigan Army Medical CenterTacoma, Washington; Department of Anesthesiology, Rush University Medical CenterChicago, Illinois; Department of Anesthesia and Critical Care, University of ChicagoChicago, Illinois; Department of Anesthesiology and Perioperative Medicine, Rutgers Robert Wood Johnscon Medical SchoolNew Brunswick, New Jersey; Department of Anesthesiology, Advocate Illinois Masonic Medical CenterChicago, Illinois; Department of Anesthesiology, Harbor-UCLA Medical CenterTorrance, California; Department of Anesthesioogy, Northwestern University Feinberg School of MedicineChicago, Illinois; Department of Anesthesiology and Pain Management, University of Texas Southwestern Medical CenterDallas, Texas; Surgical Intensive Care Unit, Massachusetts General HospitalBostonMassachusetts; one99Franklin, Massachusetts; 1 Jamie L. Sparling, M.D., Hassan S. Dashti, Ph.D., R.D., and Alexander Nagrebetsky, M.D.: Department of Anesthesiology, Mass General Brigham, Boston, Massachusetts; Department of Anaesthesia, Harvard Medical School, Boston, Massachusetts.; 2 Thomas Edmiston, M.B., B.Chir.: University of California San Francisco, Department of Anesthesia and Perioperative Care, San Francisco, California.; 3 Prashant Nasa, M.D.: Department of Anaesthesia and Critical Care Medicine, University Hospital Plymouth NHS Trust, Plymouth, United Kingdom; Department of Targeted Intervention, University College London, London, United Kingdom.; 4 Ravindra A. Gupta, M.D.: Division of Critical Care, Department of Anesthesiology, Northwestern University Feinberg School of Medicine, Chicago, Illinois.; 5 Kunal Karamchandani, M.D.: Department of Anesthesiology and Pain Management, University of Texas Southwestern Medical Center, Dallas, Texas.; 6 Ashish K. Khanna, M.D., M.S.: Department of Anesthesiology, Section on Critical Care Medicine, Wake Forest School of Medicine, Winston-Salem, North Carolina; Outcomes Research Consortium, Houston, Texas.; 7 Jarva Chow, M.D., M.S., M.P.H.: Department of Anesthesia & Critical Care, University of Chicago, Chicago, Illinois.; 8 Federico Bilotta, M.D., Ph.D.: Department of Anesthesiology, University of Rome “Tor Vergata”, Rome, Italy.; 9 Alisha Sachdev, M.D.: Department of Anesthesiology, Rush University, Chicago, Illinois.; 10 Roshni Sreedharan, M.D.: Department of Anesthesiology, Integrated Hospital Care Institute, Cleveland Clinic, Cleveland, Ohio.; 11 Shahla Siddiqui, M.B.B.S., M.Sc.: Department of Anaesthesia, Harvard Medical School, Boston, Massachusetts; Department of Anesthesiology, Beth Israel Deaconess Medical Center, Boston, Massachusetts.; 12 Paul E. Wischmeyer, M.D., E.D.I.C.: Departments of Anesthesiology and Surgery, Duke University School of Medicine, Raleigh-Durham, North Carolina.; 13 Christian Stoppe, M.D..: Department of Anaesthesiology, Intensive Care, Emergency and Pain Medicine, University Hospital Würzburg, Würzburg, Germany; Department of Cardiac Anesthesiology and Intensive Care Medicine, Deutsches Herzzentrum der Charité, Berlin, Germany; Department of Anesthesiology, Critical Care and Pain Medicine, McGovern Medical School, The University of Texas Health Science Center at Houston, Houston, Texas.; 14 Jeffrey I. Mechanick, M.D.: Kravis Center for Cardiovascular Health, Mount Sinai Fuster Heart Hospital, Icahn School of Medicine at Mount Sinai, New York City, New York.; 15 D. Dante Yeh, M.D., M.H.P.E.: Division of Emergency General Surgery, Denver Health Medical Center, Denver, Colorado; Department of Surgery, University of Colorado School of Medicine, Denver, Colorado.; 16 Ronald G. Pearl, M.D., Ph.D.: Department of Anesthesiology, Perioperative and Pain Medicine, Stanford University School of Medicine, Stanford, California.; 17 Todd W. Rice, M.D., M.Sc.: Division of Allergy, Pulmonary, and Critical Care Medicine, Vanderbilt University Medical Center, Nashville, Tennessee.; 18 Michael DeWane, M.D.: Department of Surgery, Mass General Brigham, Boston, Massachusetts; Department of Surgery, Harvard Medical School, Boston, Massachusetts.; 19 Martin Rosenthal, M.D.: Division of Acute Care Surgery, Department of Surgery, University of Florida College of Medicine, Gainesville, Florida.; 20 Richard P. Dutton, M.D., M.B.A.: US Anesthesia Partners, Fulton, Maryland.

## Abstract

In the absence of guidelines from professional societies, periprocedural fasting practices in enteral nutrition vary widely and often lead to preventable, clinically significant nutritional deficits. To address the heterogeneity of current clinical practice, the authors aimed to develop expert consensus on best practices for the periprocedural management of tube feeding using a modified Delphi consensus process. Draft recommendations were iteratively modified during a four-round Delphi process by a multidisciplinary international panel of experts. After iterative consolidation and modification, a total of 20 recommendations were produced by round 4, and all recommendations achieved stability. Final recommendations advocate for the protocolization of institutional perioperative fasting approaches and establish best practices for perioperative management of tube feeding in patients with and without a secure airway (cuffed endotracheal or cuffed tracheostomy tube) across a range of common clinical scenarios. The recommendations are intended to harmonize local practices internationally and safely minimize unnecessary periprocedural nutritional loss.

To minimize the risk of periprocedural aspiration during general anesthesia or sedation, patients are routinely fasted in advance of procedures. Aspiration, while rare, may be life-threatening. Unnecessary fasting, however, also causes harm, contributing to significant underfeeding, which is often underrecognized by clinicians.^1^ For patients with oral intake (solids or liquids) before their procedures, professional society guidelines have established a standard of care.^2–8^ However, to date, society guidelines have not addressed the periprocedural management of tube feeding, leaving individual clinicians and institutions without guidance.

Patients receiving tube feeds are frequently subjected to the same prescribed fasting duration as patients who take oral nutrition.^9^ These practices are indiscriminately extended to patients with a secure airway—such as an endotracheal tube (ETT) or cuffed tracheostomy tube—who do not experience a period of an unprotected airway between anesthesia induction and airway securement.^10^ Similarly, patients whose feeding tubes are placed in the distal small bowel experience prolonged fasting times despite decreased aspiration risk.^9,11^ Further, hospitalized patients often undergo multiple and/or repeated procedures requiring anesthesia or sedation, such as serial debridement, repeated operations for trauma, and interventional radiology procedures (fig. 1). The lack of certainty in timing of these interventions due to their “waitlist” or “add-on” nature further increases the duration of fasting due to procedures that are incrementally delayed and often deferred until the following day. Periprocedural fasting in the setting of delayed and repeated procedures commonly leads to nutritional deficits^1,12,13^ in patients who are at high risk for malnutrition, and in whom fasting may be unnecessary for procedures with low aspiration risk. Underfeeding may aggravate the loss of muscle mass and affect other mid- and long-term outcomes including mortality, length of stay (LOS), and incidence of infection.^14^

**Fig. 1.**
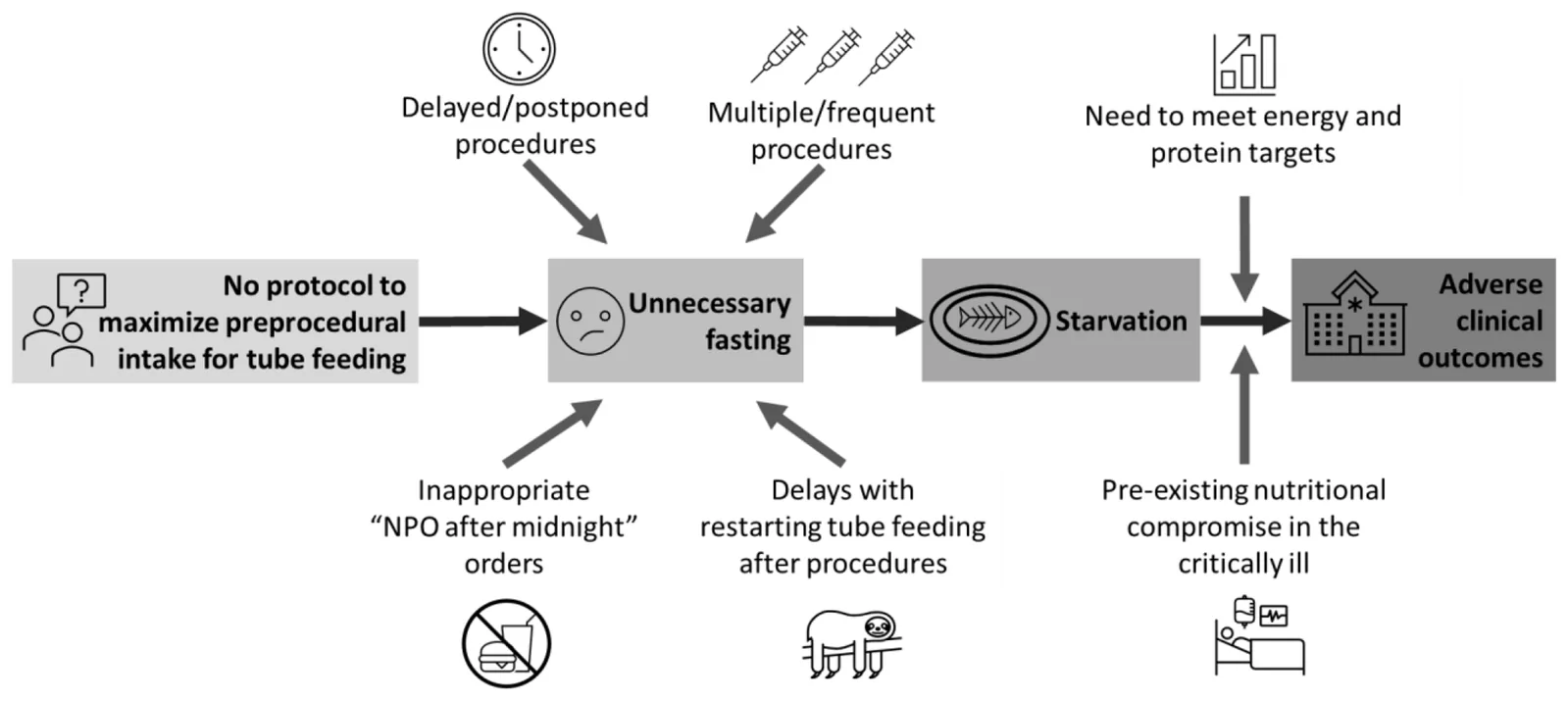
Consequences of the lack of protocols to minimize interruptions in nutrition delivery among perioperative and procedural patients receiving tube feeding. NPO, nil per os.

The practice of prolonged fasting to reduce the risk for aspiration must be balanced against the risks of malnutrition, which is a significant prognostic factor for morbidity, mortality, sarcopenia, loss of muscle mass, and LOS in hospitalized patients.^15–19^ Malnutrition is associated with higher infection rates, increased LOS, higher readmission rates, muscle wasting, pressure ulceration, worse functional outcomes, increased fall risk, higher treatment costs, and increased mortality.^16,20^ Avoidable interruptions of tube feeding among surgical patients are associated with longer intensive care unit (ICU) and hospital LOS and higher rates of infectious and overall complications.^1,21^

In the absence of guidance from professional societies, institutional periprocedural fasting practices in enteral nutrition vary widely^9^ and often lead to preventable, clinically significant nutritional deficits.^12,22^ To address the heterogeneity of current clinical practice, we aimed to develop an expert consensus on best practices for the periprocedural management of tube feeding.

## Modified Delphi Process

We followed a modified Delphi process to develop clinical practice recommendations (fig. 2) and used the Accurate Consensus Reporting Document (ACCORD) as a framework for this study.^23^ The study was approved by the Mass General Brigham Human Research Protection Program (protocol 2025P000937).

**Fig. 2.**
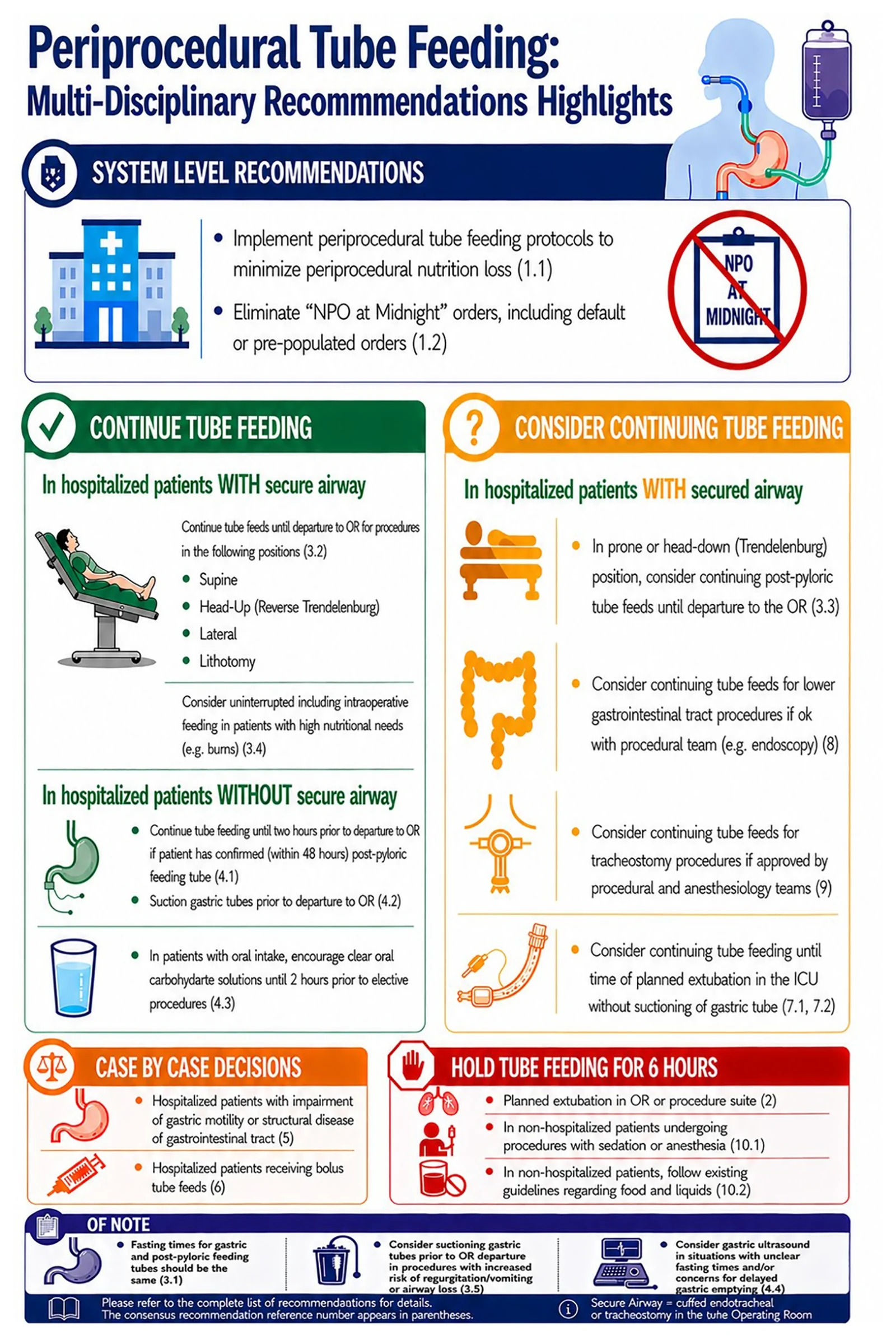
Summary of the expert consensus recommendations derived from a four-round modified Delphi process.

### Team Structure

We assembled two teams (fig. 3): (1) The Task Force on Periprocedural Fasting in Patients Who Receive Tube Feeding (n = 29) and (2) the Delphi Expert Panel (n = 11). Within the Task Force, a Steering Committee (n = 11: A.N., J.L.S., T.E., K.K., P.N., F.B., A.S., J.C., R.S., S.S., A.K.K.) coordinated the Delphi process. The Steering Committee consisted of 91% (10 of 11) attending anesthesiologists-intensivists, and one incoming anesthesiology resident physician. Most Steering Committee members (8 of 11) were a part of the American Society of Anesthesiologists (ASA; Schaumburg, Illinois) Committee on Critical Care Medicine (COCCM), including leaders and contributors to the previous ASA COCCM practice recommendations. This project was unfunded.

**Fig. 3.**
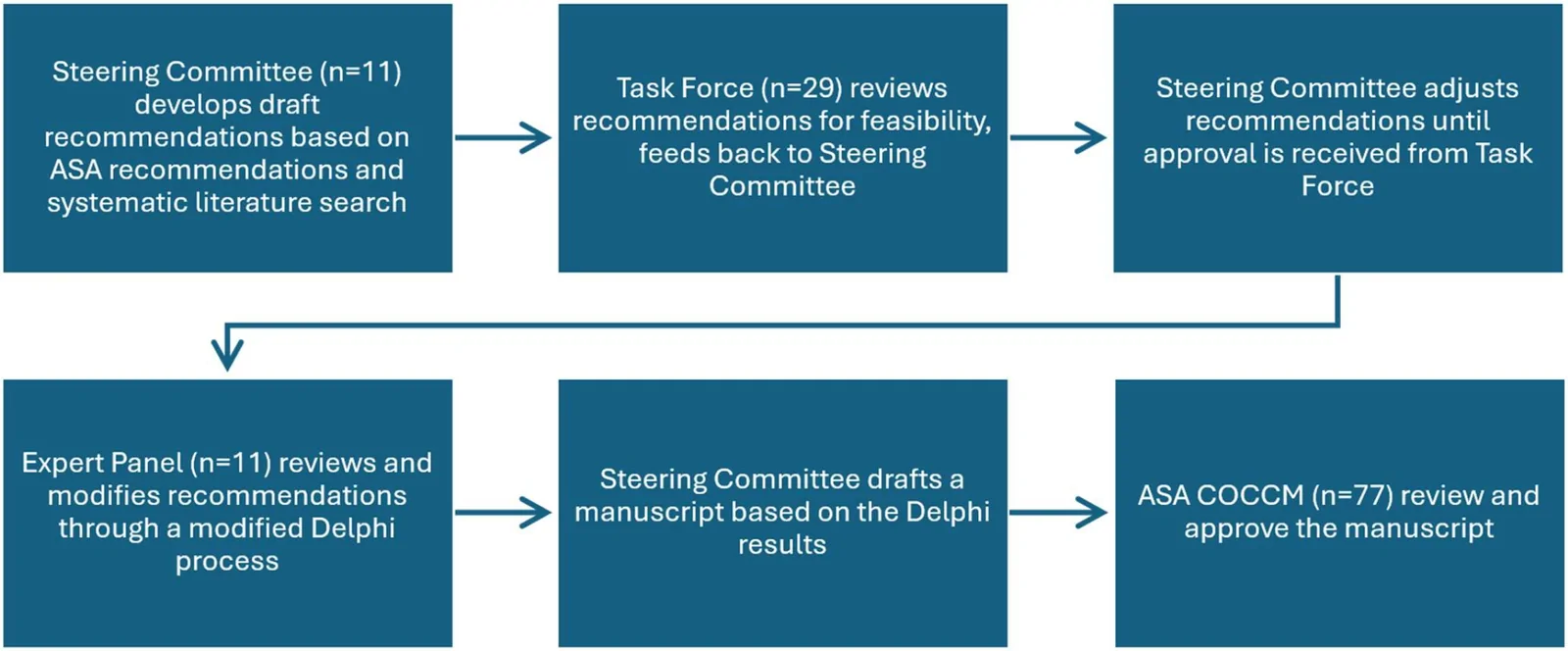
A modified Delphi process to develop clinical practice recommendations. ASA, American Society of Anesthesiologists; COCCM, Committee on Critical Care Medicine.

### Creation of Clinical Practice Recommendations

The Steering Committee reviewed the previous ASA COCCM Recommendations (fig. 3)^24^ along with the results of a systematic literature search on periprocedural fasting conducted by another group^25^ and funded by the European Society of Anesthesiology and Intensive Care (with permission from the authors). The findings were used to draft a series of initial clinical practice recommendations that were presented to the Task Force (fig. 3), composed of 26 anesthesiologists-intensivists from the ASA COCCM, a critical care nurse, and a patient-stakeholder.

### Expert Panel Selection

The Task Force assessed the draft clinical practice recommendations for feasibility in a clinical setting, and following the Task Force’s approval, the recommendations were sent to the content experts as part of the first round of the modified Delphi process. The Expert Panel, separate from the Steering Committee and the Task Force, included 11 clinicians who met eligibility criterion 1 plus at least one of criteria 2, 3, or 4:

1. Significant clinical experience in periprocedural/critical illness nutrition (at least 5 yr).
2. Authorship in at least one peer-reviewed publication on periprocedural/critical illness nutrition.
3. Participation in the development of local/regional/national recommendations on periprocedural/critical illness nutrition.
4. Advocacy/leadership/fellowship (certification/accreditation) in enteral/parenteral nutrition professional societies.

Using the eligibility criteria, panelists were purposively identified from the investigators’ professional networks and invited *via* direct email, with centralized oversight of invitations. Before initiation, additional panelists were recruited through targeted referrals to address gaps in expertise; all 11 invited individuals accepted participation. The Task Force members, including the Steering Committee, did not participate in the Delphi survey.

### Delphi Process

The Delphi process is a structured method for achieving consensus among a panel of experts through a series of iterative questionnaires, with core elements including selection of an expert panel, anonymous response, controlled feedback between rounds, repeat rounds allowing reconsideration, and a predefined method for determining consensus. This study was modified from the original Delphi methodology in that the experts were presented with candidate consensus recommendations developed from a comprehensive review of the literature, existing guidelines, and expert input in the first round, replacing a traditional open-ended first round. This modification improved efficiency and helped to ensure that important concepts identified from the literature are not omitted. The modification does not, however, diminish the validity of the study as the defining elements of the Delphi process are retained.^26^ The recommendations were distributed *via* email as a Microsoft Excel (MS Office 2019, Microsoft Corp, Redmond, Washington) spreadsheet to the experts. Before distribution, the instrument was piloted by members of the Steering Committee. Identities of the experts remained anonymous until the end of the Delphi process to avoid bias or group pressure. Responses were collected on a seven-point Likert scale, with the ability to provide comments for each recommendation or suggest additional recommendations. Based on feedback, recommendations were modified or removed for subsequent rounds by the Steering Committee. The Delphi rounds were conducted from May 2025 to November 2025. The Steering Committee also distributed the results of each round in a summarized and anonymized format among the experts.

### Consensus and Stability

For recommendations, consensus was defined as 70% or greater agreement (scores 5 to 7) or disagreement (scores 1 to 3). Where 90% or greater of responses were scores of 5 to 7 or 1 to 3, we defined this as strong agreement or disagreement. Medians (interquartile range) were used to describe the central tendency and dispersion of responses. Stability of responses between rounds was assessed using a nonparametric chi-square (*χ*^2^) test or the Kruskal–Wallis test from round 2 onwards.^26^ A *P* value less than 0.05 was considered a significant variation, or unstable. Microsoft Excel was used for statistical analysis. Recommendations were included in the Delphi rounds until the criteria for stability were reached.

The Steering Committee drafted expert clinical practice recommendations based on recommendations that achieved consensus during the Delphi process. The results of the Delphi process, clinical practice recommendations, and manuscript were circulated among the members of the ASA COCCM for review. Approval was required from at least 90% of clinician-members of the ASA COCCM before finalization and publication of these practice recommendations.

### Consensus Development

We sent email invitations to 11 experts, all of whom agreed to participate and voted in all four Delphi rounds. The expert panel included an international group of five anesthesiologists, three surgeons, a pulmonologist-intensivist, an endocrinologist, and a registered dietitian nutritionist (Supplemental Digital Content1, table 1: https://links.lww.com/ALNO/A29).

The first modified Delphi round included 28 draft recommendations, based on round 1 voting and feedback (Supplemental Digital Content 1, tables 2 and 3: https://links.lww.com/ALNO/A29); these draft recommendations were consolidated into 20 recommendations. In round 2, all except one of the 20 recommendations achieved consensus. After modification of the recommendations that did not achieve consensus based on expert feedback, all 20 recommendations entered round 3 and achieved consensus. The 19 unmodified recommendations reached stability with agreement ranging from 82 to 100% in round 3 (Supplemental Digital Content 1, table 2: https://links.lww.com/ALNO/A29). Ten recommendations achieved consensus with 100% agreement in round 3. The recommendations modified between round 2 and round 3 had stability confirmed during round 4.

The final clinical practice recommendations (table 1) were circulated to the Task Force (n = 28) and the ASA COCCM (n = 77) for approval. The Task Force demonstrated a 100% response rate with unanimous endorsement, and the ASA COCCM unanimously endorsed the recommendations. Figure 2 summarizes the final recommendations.

**Table 1.** Clinical Practice Recommendations Derived from a Four-round Modified Delphi Process

Patient Population	Clinical Practice Recommendation
Hospitalized patients	*1. Clinical practice recommendations for institutions*
	1.1 Institutions should implement periprocedural tube feeding protocols based on national recommendations to minimize periprocedural nutrition loss. 1.2 Institutions should eliminate “NPO after midnight” orders, including default or pre-populated orders.
	*2. Planned postprocedure extubation*
	2. Patients who will be extubated after the procedure should follow conventional fasting guidelines (*i.e.*, 6 h for most tube feeds).
	*3. Patients with a secure airway*
	In hospitalized patients WITH a secure airway (cuffed endotracheal tube or cuffed tracheostomy tube) who undergo procedures with sedation or anesthesia that do not require fasting for surgical indications* or involve airway instrumentation: 3.1 Fasting times for both gastric and postpyloric feeding tubes should be the same. 3.2 For procedures performed primarily in the supine, head-up (reverse Trendelenburg), lateral, or lithotomy positions, tube feeding *via* gastric or postpyloric tubes should continue until departure to the operating room. 3.3 For patients with a postpyloric feeding tube having procedures in prone or head-down (Trendelenburg) position, consider continuing tube feeding until departure to the operating room. 3.4 Consider uninterrupted periprocedural tube feeding *via* gastric or postpyloric feeding tube, including continuing tube feeding during the procedure, especially in patients with high nutritional needs, such as burn patients. 3.5 Consider suctioning gastric tubes or gastric ports of combined tubes before departure to the operating room in procedures with increased risk of regurgitation/vomiting (*e.g.*, abdominal insufflation) or airway loss (*e.g.*, craniofacial procedures).
	*4. Patients without a secure airway*
	In hospitalized patients WITHOUT a secure airway (cuffed endotracheal tube or cuffed tracheostomy tube) who undergo procedures with sedation or anesthesia that do not require fasting for surgical indications* or involve airway instrumentation:† 4.1 Continue tube feeding until 2 h before departure to the operating room if a patient has a surgically placed or radiologically confirmed (within 48 h before procedure) postpyloric feeding tube. 4.2 Suction gastric feeding tube if it permits suctioning before departure to the operating room. 4.3 In hospitalized patients who maintain oral intake in addition to tube feeding, clear oral carbohydrate solutions should be encouraged until 2 h before elective procedures. 4.4 Consider gastric ultrasonography by a trained provider in cases with uncertain fasting time and/or particular concerns for delayed gastric emptying.‡
	*5. High aspiration risk*
	5. In hospitalized patients with documented or diagnosed major impairment of gastrointestinal motility or significant structural disease of the gastrointestinal tract, duration of periprocedural fasting should be determined on a case-by-case basis, following a discussion with the procedural, anesthesiology, and intensive care teams.‡
	*6–9. Special considerations*
	6. There are insufficient data to guide periprocedural fasting in hospitalized patients receiving bolus tube feeding, irrespective of the presence of a secure airway. Decisions regarding the optimal duration of periprocedural fasting in these patients should therefore be made on a case-by-case basis.
	7.1 Consider continuing tube feeding until the time of planned extubation in the ICU. 7.2 Continued tube feeding until the time of planned ICU extubation may be implemented without suctioning/sumping (if Salem Sump tube) the stomach before extubation.
	8. Patients with a secure airway undergoing lower GI tract procedures (*e.g.*, endoscopy, bowel surgery) may continue to receive enteral nutrition until the time of procedure if approved by the procedural team.
	9. Patients with a secure airway undergoing tracheostomy may continue to receive tube feeding until the time of procedure if approved by the procedural and anesthesiology teams.
Nonhospitalized patients	*10. Nonhospitalized patients*
	In nonhospitalized patients who undergo procedures with sedation or anesthesia: 10.1 Tube feeding should be held 6 h before onset of sedation or anesthesia. 10.2 Fasting for food and liquids should follow existing periprocedural fasting guidelines.

* Examples: bowel resection, enteroscopy. †Airway instrumentation includes tracheal intubation, tracheal extubation, airway exchange, and other procedures involving the airway. ‡Examples of major motility impairment and structural diseases include gastroparesis, bowel obstruction, ileus, recent administration of glucagon-like peptide-1 receptor agonist (GLP-1RA), compromised lower esophageal sphincter, and gastrointestinal bleeding. GI, gastrointestinal; ICU, intensive care unit; NPO, nil per os.

## 1. Clinical Practice Recommendations for Institutions

1.1 *Institutions should implement periprocedural tube feeding protocols based on national recommendations to minimize periprocedural nutrition loss.*
1.2 *Institutions should eliminate “NPO (nil per os) after midnight” orders, including default or prepopulated orders.*

Periprocedural fasting practices for patients who receive tube feeding are commonly defined at an institutional level using local guidelines and protocols.^9,10^ The experts suggested institutional protocolization to facilitate implementation of the identified best practices. Incremental implementation of an institutional protocol with multidisciplinary involvement and the use of visual guides has optimized periprocedural fasting practices in the general surgical population.^27^ Notably, the recommendation to protocolize periprocedural tube feeding received unanimous agreement in rounds 2 and 3 (Supplemental Digital Content 1, table 2: https://links.lww.com/ALNO/A29), indicating firm support among our multidisciplinary team. Experts expressed support for this recommendation, commenting that it is “effective and evidence-based.”

Our clinical practice recommendations support the ongoing efforts^28^ to prevent periprocedural fasting after midnight as a standard practice. Such a practice has been widely criticized as unnecessary and likely harmful.^29,30^ The clinical practice recommendation to eliminate “NPO after midnight” orders achieved 100% agreement in round 3 (Supplemental Digital Content 1, table 2: https://links.lww.com/ALNO/A29). Our original recommendation to minimize rather than eliminate such orders was modified based on input from two Delphi experts, who suggested that instead of minimizing, these orders should be eliminated. “Instead of ‘minimize’ it could even be stronger” and argued to “… eliminate these orders.*”*

## 2. Planned Postprocedure Extubation

*2. Patients who will be extubated after the procedure should follow conventional fasting guidelines (i.e., 6 h for most tube feeds).*

Planned extubation immediately after the procedure changes clinical decision-making with respect to preoperative fasting duration. Theoretically, patients would be extubated at the end of the procedure once able to protect their airway. A recent randomized controlled trial comparing continuing enteral nutrition until extubation in critically ill patients to a strategy of a 6-h fast with accompanying gastric tube suctioning found that the continued feeding strategy was noninferior in terms of extubation failure within 7 days.^31^ However, positioning-related (*e.g.*, head of bed not elevated to 30 degrees as is commonly done in the ICU) or other physiologic changes in the operating room or procedural area may increase the risk of passive regurgitation in patients who would no longer have a protected airway. The recommendation was initially assessed with the example of an 8-h fast for most tube feed formulas, and the expert panel expressed 64% agreement in round 1. In round 2, the example fasting time was shortened to 6 h based on expert feedback and consensus was achieved with 91% agreement in round 2, confirmed with 91% agreement in round 3.

## 3. Patients with a Secure Airway

In hospitalized patients WITH a secure airway (cuffed endotracheal tube or cuffed tracheostomy tube) who undergo procedures with sedation or anesthesia that do not require fasting for surgical indications (e.g., bowel resection, enteroscopy) or involve airway instrumentation:

3.1 *Fasting times for both gastric and postpyloric feeding tubes should be the same.*
3.2 *For procedures performed primarily in the supine, head-up (reverse Trendelenburg), lateral, or lithotomy positions, tube feeds via gastric or postpyloric tubes should continue until departure to the operating room.*
3.3 *For patients with a postpyloric feeding tube having procedures in prone or head-down (Trendelenburg) position, consider continuing tube feeding until departure to the operating room.*
3.4 *Consider uninterrupted periprocedural tube feeding via gastric or postpyloric feeding tube, including continuing tube feeding during the procedure, especially in patients with high nutritional needs, such as burn patients.*
3.5 *Consider suctioning gastric tubes or gastric ports of combined tubes before departure to the operating room in procedures with increased risk of regurgitation/vomiting (e.g., abdominal insufflation) or airway loss (e.g., craniofacial procedures).*

### Gastric *versus* Postpyloric Feeding Tubes

Although postpyloric feeding tubes have been shown to provide clinical benefit compared with gastric feeding tubes in terms of pulmonary and gastrointestinal complications, gastric feeding can typically be initiated significantly sooner than postpyloric feeding due to delay in achieving postpyloric placement.^11,32^ Further, pulmonary aspiration rates detected by pepsin present in tracheal aspirates were not different in ICU patients in the prone position receiving gastric compared with postpyloric feeds.^33^ In the absence of a demonstrable benefit in avoiding aspiration, the expert panel expressed 64% agreement on Recommendation 3.1 in round 1. Further discussion of the rationale for this recommendation: (1) regurgitation or vomiting is unlikely to cause aspiration due to the presence of a secure airway, and (2) it is the secure airway, rather than tube location, that conveys safety, resulted in an increase to 91% agreement in round 2 and 100% in round 3. One expert pointed out that there should be “exceptions for gastroparesis or pyloric outlet obstruction,” which is endorsed in Recommendation 5.

### Patient Position

Taken together with other feeding strategies, continuing tube feeding until the time of departure to the operating room can increase energy and protein intake in ICU patients and was associated with a significant decrease in pneumonia in one study.^34^ In round 1, Recommendation 3.2 was assessed separately for each position, with the expert panel expressing 91% agreement with the supine position, 90% for the head-up (reverse Trendelenburg) position, 80% for the lateral position, and 90% for the lithotomy position. One expert commented that after feeding countless proned patients during the COVID-19 pandemic, they are now “agnostic to position in regard to enteral feeds in those with secured airways.” Accordingly, these positions were combined into one recommendation, reaching 91% agreement in round 2 and 100% agreement in round 3.

In critically ill patients with a secure airway, gastric residual volumes are similar between the supine and prone positions,^35^ and one study of intra-operative feeding in burn patients found no significant differences in the rate of pneumonia in patients receiving tube feeds in the prone position.^36^ In round 1, 60% of experts agreed with continuing tube feeds until the time of departure to the operating room (OR) for patients in the head-down (Trendelenburg) position and 50% agreed for the prone position. These recommendations were combined in round 2 and agreement with the recommendation increased to 73%, with a further increase to 91% in round 3. One expert commented that there “could be some concern in head-down position for airway loss or increased risk of massive aspiration, especially if insufflation of abdomen is used, but would still consider with appropriate airway protection precautions.”

### Uninterrupted Periprocedural Feeding

A number of systematic reviews and several observational studies reported that intraoperative tube feeding increases delivered calories and may reduce energy deficits, without a clear increase in aspiration, and accordingly, major nutrition societies emphasize early and continuous enteral nutrition in patients with high-energy need and support of individualized continuation of feeds in the periprocedural period when clinically appropriate.^37–39^ The strength of evidence is reflected by 91% agreement in round 1 and 100% agreement in rounds 2 and 3. Expert comments reflect that uninterrupted periprocedural feeding “is established for burn patients,” and that it applies “especially for burn patients having several smaller surgical procedures and a significantly elevated energy expenditure.” Others commented, however, that intraoperative feeding “may not be appropriate for prolonged procedures under anesthesia,” and that they “agree with patients with high nutritional needs (burn),” while suggesting that with the current available literature it is difficult to recommend “routine intra-operative continuation.”

### Gastric Suctioning

Many centers routinely suction feeding tubes before departure to the operating room, regardless of planned surgical position. Initially, experts were asked separately whether they support preoperative suctioning for gastric tubes (60% agreement), postpyloric (0% agreement), and gastric in addition to postpyloric or combined tubes (80% agreement). These recommendations were combined to reflect “suctioning gastric tubes or gastric ports of combined tubes” and specifically for procedures with risks of regurgitation, vomiting, or airway loss. With this modification, Recommendation 3.5 achieved 91% agreement in round 2 and 100% in round 3.

## 4. Patients without a Secure Airway

In hospitalized patients WITHOUT a secure airway (cuffed endotracheal tube or cuffed tracheostomy tube) who undergo procedures with sedation or anesthesia that do not require fasting for surgical indications(e.g., bowel resection, enteroscopy) or involve airway instrumentation (airway instrumentation includes tracheal intubation, tracheal extubation, airway exchange, and other procedures involving the airway):

4.1 *Continue tube feeding until 2 h before departure to the operating room if a patient has a surgically placed or radiologically confirmed (within 48 h before procedure) postpyloric feeding tube.*
4.2 *Suction gastric feeding tube if it permits suctioning before departure to the operating room.*
4.3 *In hospitalized patients who maintain oral intake in addition to tube feeding, clear oral carbohydrate solutions should be encouraged until 2 h before elective procedures.*
4.4 *Consider gastric ultrasonography by a trained provider in cases with uncertain fasting time and/or particular concerns for delayed gastric emptying (examples of major motility impairment and structural diseases include gastroparesis, bowel obstruction, ileus, recent administration of glucagon-like peptide-1 receptor agonist, compromised lower esophageal sphincter, and gastrointestinal bleeding).*

Suctioning *in situ* gastric tubes reduces gastric volume and therefore theoretically lowers aspiration risk. This is an accepted practice in published tube feeding protocols as a practical mitigation strategy for patients without a secure airway. While this recommendation is frequently cited in reviews and clinical guidelines on aspiration prophylaxis,^40–42^ direct evidence supporting this practice is limited. This recommendation reached consensus with 100% agreement in round 1 and 91% agreement in rounds 2 and 3.

### Postpyloric Feeding Tubes

Feeding tubes ending in the small bowel, either surgically implanted jejunostomy tubes or nasoduodenal/ nasojejunal tubes with a confirmed postpyloric tip location, theoretically incur minimal risk of aspiration. One prospective observational cohort study compared patients receiving jejunostomy feeds until transport to the OR with patients who were fasted for 8 h before transport. There were no episodes of aspiration, and patients with the shortened fast received more calories on the day of surgery and the first day following surgery.^43^ This recommendation reached consensus with 100% agreement in rounds 1 and 2 and 91% agreement in round 3, although one expert commented that one “may want to note the caveats”—including the initial guidance that the recommendations as a whole do not apply to patients with major impairment of gastrointestinal motility or significant structural disease of the gastrointestinal tract (*e.g.*, gastroparesis, bowel obstruction, ileus).

### Oral Intake

Evidence supporting the continuation of clear liquid carbohydrate solutions until 2 h before elective procedures is robust. A 2003 Cochrane review of 22 randomized controlled trials concluded that there was no evidence to suggest that a shortened fast for clear liquids increased the risk of aspiration, regurgitation, or related morbidity and that a shortened fasting time for clear liquids was associated with significantly *lower* gastric residual volumes.^44^ While studies specifically in patients with an *in situ* feeding tube are lacking, experts expressed 91% agreement in round 1, and 100% agreement in both rounds 2 and 3, commenting that the recommendation “fits with ERAS (Enhanced Recovery After Surgery).”

### Gastric Ultrasound

Gastric ultrasound is an increasingly utilized modality for stratifying patients’ aspiration risk based on the quantity and quality of gastric content. A recent review of five prospective, observational studies of 793 patients showed that between 18 and 56% of patients presenting for emergency surgery are found to have a “full stomach” using gastric ultrasound at the time of induction.^45^ The American Society of Regional Anesthesia and Pain Medicine conducted a narrative review of gastric ultrasound for regional anesthesia and pain medicine procedures and concluded that gastric ultrasound use was supported for patients in active labor, those undergoing urgent cesarean sections, and those with diabetes, with conditional support for obesity, emergency care, enteral feeding, and glucagon-like peptide-1 receptor agonist use.^46^ Experts were initially mixed in their responses, with 64% agreement in round 1, and comments such as “would be helpful to understand the data for the usefulness of ultrasound in detecting full stomach and ultimate risk of aspiration.” Concerns were also raised that findings with gastric ultrasound may be “very dependent on the provider doing the ultrasound.” With additional clarification that the intent of the gastric ultrasound is not to replace fasting, but to supplement it, the recommendation reached consensus with 70% agreement in round 2 and 82% agreement in round 3.

## 5. High Aspiration Risk

5. *In hospitalized patients with documented or diagnosed major impairment of gastrointestinal motility or significant structural disease of the gastrointestinal tract, duration of periprocedural fasting should be determined on a case-by-case basis, following a discussion with the procedural, anesthesiology, and intensive care teams (examples of major motility impairment and structural diseases include gastroparesis, intestinal obstruction, ileus, compromised lower esophageal sphincter, gastrointestinal bleeding).*

The existing literature base does not cover every situation for patients with impaired gastrointestinal motility or structural disease of the gastrointestinal tract. In these unique situations, the expert panel reached consensus with 100% agreement in round 1, 91% agreement in round 2, and 100% agreement in round 3. However, one expert recognized that “These types of discussion are very frustrating [because] they are driven by very little scientific evidence and delay cases for zero to no reason.” This recommendation is aligned with existing fasting recommendations that stipulate that they apply to healthy patients and may need modification based on clinical judgement^2^ and that decisions must be individualized.^47^

## 6-9. Special Considerations

### Bolus Tube Feeding

6. *There are insufficient data to guide periprocedural fasting in hospitalized patients receiving bolus tube feeding, irrespective of the presence of a secure airway. Decisions regarding the optimal duration of periprocedural fasting in these patients should therefore be made on a case-by-case basis.*

Bolus tube feeding offers several potential benefits over continuous enteral nutrition. Bolus gastric feeds may better stimulate gut hormone response, including pancreatic enzymes and incretins, and enhance gastric emptying, maintaining gut integrity and reducing ileus. Bolus tube feeds have been associated with higher energy and protein delivery and faster time to goal volume.^48^ Some studies have shown metabolic benefits including improved glycemic control and tolerance in critically ill patients,^49^ although data are mixed.^50^ Bolus tube feeds may also reduce cost and improve mobility in patients at home or in long-term care facilities, as they do not require an infusion pump, permit normal daytime activities, and are less resource intensive. Finally, bolus tube feeding is thought to promote cyclic gastrointestinal loading, which may aid the return of normal feeding patterns during rehabilitation. However, to the authors’ knowledge, optimal preoperative fasting duration has not been studied for bolus tube feeds. Accordingly, the expert panel expressed agreement with Recommendation 6 in each of the rounds (91% agreement in round 1, 82% agreement in round 2, and 91% agreement in round 3). One expert commented, “my clinical experience indicates this should also be safe for same guidelines as continuous feeds,” while another indicated “should simply wait the 6-8 hours of fasting” for bolus tube feeds.

### Continuation of Tube Feeding

7.1 *Consider continuing tube feeding until the time of planned extubation in the ICU.*
7.2 Continued *tube feeding until the time of planned ICU extubation may be implemented without suctioning/sumping (if Salem Sump tube) to the stomach before extubation.*

A recent randomized controlled trial in over 1,000 patients compared continued enteral nutrition with fasting and feeding tube suctioning before planned extubation in the ICU. The authors found continued enteral nutrition to be noninferior on the primary outcome of extubation failure within 7 days of extubation, with a rate of 17.2% in the continued enteral nutrition group *versus* 17.5% in the fasted group, and a lower rate of pneumonia at 14 days in the continued enteral nutrition group, 1.6 *versus* 2.5%.^31^ Unlike patients experiencing a planned procedure before extubation, positional and other physiologic changes are unlikely to prompt passive regurgitation, and the patients would presumably be extubated once awake and able to protect their airway. The expert panel expressed 82% agreement with Recommendation 8 during round 1. In round 2, language was softened to “consider…” and, with this amendment, the recommendation reached 91% agreement in round 2 and 100% agreement in round 3. One expert commented that, “Strong evidence [is] available,” and cited the above study. Another expert agreed with the recommendation for all feeding tubes, but emphasized, “especially if post-pyloric.” A separate expert expanded that, “…I only hold tube feeds in patients I am extubating if I think there is a high risk of reintubation.” Conversely, Recommendation 7.2 did not reach consensus in round 1, with only 36% of experts in agreement. One expert commented, “If available to be suctioned, I would still suction the nasogastric tube prior to planned ICU extubation.” However, in round 2, consensus was reached with 82% agreement, rising to 91% agreement in round 3. This may be explained by increasing expert familiarity with the above-mentioned clinical trial.^31^

8. *Patients with a secure airway undergoing lower gastrointestinal (GI) tract procedures (e.g., endoscopy, bowel surgery) may continue to receive enteral nutrition until the time of procedure if approved by the procedural team.*

In one recent international, multicenter, double-blind, randomized controlled trial of patients undergoing elective colorectal surgery with primary anastomosis, patients were randomized to receive lipid-rich enteral nutrition *via* nasojejunal tube from 3 h before surgery to 6 h after surgery or to no periprocedural nutrition.^51^ There was a nonsignificant increase in the rate of pneumonia in the enteral nutrition (8 *vs.* 2%, RR 1.06; 95% CI: 1.00 to 1.12), which is worthy of more study. While the trial did not show improvement in the primary outcome of postoperative ileus, it does demonstrate that it is feasible to feed patients even during lower GI tract procedures and was the basis for Recommendation 10. The expert panel expressed 64% agreement in round 1, which rose to 73% in round 2 and 91% in round 3. One expert, however, commented that they were “still personally unsure” while another mentioned that “in these situations the use of supplemental parenteral nutrition might make sense.”

9. *Patients with a secure airway undergoing tracheostomy may continue to receive tube feeding until the time of procedure if approved by the procedural and anesthesiology teams.*

Bedside tracheostomy is a procedure that may involve a short period of time with an unprotected airway but is typically without positional changes or procedural perturbations that would increase the risk of aspiration in patients with a preexisting protected airway. Fasting practices for this common and routine procedure differ across and even within institutions, reflecting limited available evidence evaluating this practice.^52^ In one study of intubated ICU patients planned to undergo bedside tracheostomy, 24 patients were randomized to a 6-h fast for tube feeds compared with a 45-min fast. No patients aspirated, and one patient in the control group developed pneumonia. Length of fast was shorter and energy delivery greater in the intervention group.^53^ An additional study compared patients undergoing percutaneous tracheostomy who were fasted less than 1 h, 1 to 4 h, 5 to 12 h, and greater than 12 h; there were no differences in rates of aspiration pneumonia between groups, and groups in the longest fasting group had a higher FiO_2_ 48 h after tracheostomy compared with those who were fasting less than 1 h (47.7 *vs*. 39.1%; *P* = 0.001).^54^ The expert panel expressed 64% agreement in round 1 but achieved consensus with 73% agreement in round 2 and 91% agreement in round 3.

## 10. Nonhospitalized Patients

In nonhospitalized patients who undergo procedures with sedation or anesthesia:

10.1 *Tube feeding should be held 6 h before onset of sedation or anesthesia,*
10.2 *Fasting for food and liquids should follow existing periprocedural fasting guidelines.*

Nonhospitalized patients were considered separately as these patients are not as nutritionally vulnerable as hospitalized patients and are subject to fewer feeding interruptions. Further, few of these patients arrive with a secure airway, and delivery of their baseline tube feed formulae may be logistically difficult in the preoperative holding area compared with an inpatient floor or ICU. Recommendations 10.1 and 10.2 were initially assessed together with 82% agreement in round 1. Based on expert feedback, the recommendations were split in round 2. Recommendation 10.1 was initially assessed for an 8-h fast and had 55% agreement in round 2, with several experts favoring a 6-h fast for tube feeds, resulting in 100% agreement in round 3, confirmed in round 4. Similarly, the expert panel agreed that fasting for food and liquids should follow existing periprocedural fasting guidelines for nonhospitalized patients, with 91% agreement in both rounds 2 and 3.^2,47^

## Discussion

In this modified Delphi process–based study, a Task Force convened by the ASA COCCM assembled an international multidisciplinary team of experts in periprocedural nutrition and produced consensus clinical practice recommendations (table 1, fig. 2) on periprocedural fasting for patients who receive tube feeding. In the absence of relevant practice guidelines, this work offers national-level guidance on best clinical practices for institutions and individual clinicians. The modified Delphi process highlighted the need for institutional protocolization of periprocedural management of tube feeding to minimize unwarranted fasting, which exacerbates nutritional deficit in vulnerable patients receiving enteral nutrition.

To the authors’ knowledge, this is the first study to rigorously develop consensus-based clinical practice recommendations to address periprocedural fasting in patients receiving tube feeding, which are specifically excluded from existing periprocedural fasting guidelines.^2^ This document identifies objectives for systems-level implementation efforts to prevent the exacerbation of nutritional compromise in areas where expert consensus exists and is supported by evidence. In addition, this study proposes the groundwork for addressing the lack of high-grade evidence in this field.

The invited content experts participated in all study rounds and reached a strong agreement (90% or greater) on all but one (Recommendation 4.4) practice recommendations. Such a high level of agreement contrasts with highly heterogeneous local practices,^11^ which will likely pose a barrier to the implementation of these practice recommendations. However, the lack of national-level guidance was likely a major cause of the disparate local practices in the periprocedural management of tube feeding. Our work, therefore, represents a substantial contribution to the harmonization of periprocedural fasting practices in patients who receive tube feeding. These practice recommendations formalize at a national level existing expert consensus in areas where current literature may inform practice but is not yet definitive and validate local efforts to reduce unnecessary fasting in nutritionally vulnerable patients at the hospital and hospital system level.

While the expert panel agreed with practice recommendations intended to reduce periprocedural nutrition loss, they did uphold more conservative fasting thresholds in situations with increased risk of aspiration and/or airway loss. For example, Recommendation 3, continuing enteral nutrition until departure to the OR in patients with a secure airway, was initially assessed in each of the following positions: supine, reverse Trendelenburg (head up), Trendelenburg (head down), prone, lateral, and lithotomy. Consensus was reached in round 1 for supine, reverse Trendelenburg, lateral, and lithotomy positions, while experts disagreed with the recommendation for the prone and Trendelenburg positions, leading to Recommendation 3.3, which limited the recommendation to postpyloric feeding tubes and endorsed softer language to “Consider…” Similarly, for bolus tube feeding regimens, experts agreed there was insufficient literature to guide periprocedural fasting practices and endorsed that decisions should be made on a case-by-case basis (Recommendation 6).

## Strengths and Limitations

Strengths of the current study include its rigorous consensus-based Delphi methodology, as well as the high level of expertise and diversity of medical specialties included in our expert panel. In addition, the close involvement of many practicing critical care clinicians on the Steering Committee and the ASA COCCM bases the proposed recommendations in sound critical care practice. Importantly, the final approval for these practice recommendations by the ASA COCCM also conveys the support of practicing anesthesiologists—experts in airway management and prevention of periprocedural pulmonary aspiration. Finally, the initial draft clinical practice recommendations included in the modified Delphi process were informed by the results of a systematic literature search and were assessed for feasibility by a Task Force of practicing anesthesiologist intensivists.

Limitations include small-scale involvement of patients—only one patient with a lived experience of critical illness was included in the Task Force. We intentionally did not include the public in this study due to the complexity of the topic. We acknowledge that one of the limitations of Delphi process–based consensus recommendations, including ours, is that they may not be fully supported by evidence and reflect expert opinion on topics where literature is lacking or not robust. Hence, institutions should consider their local practices and resources while they incorporate these recommendations into practice. While we shared results from the Delphi rounds only after consensus had been achieved to minimize peer pressure, some experts may have felt pressure to align with the recommendations that continued to be assessed in subsequent rounds (group conformity). Additionally, the expert panel was not demographically and geographically diverse, with most experts practicing in the United States, and without representation of lower- and lower-middle-income countries. Although current fasting practices may vary considerably worldwide, the entire spectrum of preoperative fasting practices is represented in the United States.^9^ Third, several of the clinical practice recommendations, for example, Recommendation 5 for patients with “documented or diagnosed major impairment of gastrointestinal motility or significant structural disease of the gastrointestinal tract,” still rely on individualized clinician judgment, which is the current state for many preoperative fasting recommendations. Finally, only adult patients were considered, and the consensus recommendations therefore are limited to adult patients. This study also did not formally assess the certainty of the underlying evidence.

The task force recognizes that large-scale randomized trials are lacking to support these consensus recommendations. However, because the incidence of clinically significant aspiration is low, such trials may not be feasible. Therefore, the true impact of these recommendations on aspiration risk remains to be determined. Nevertheless, the adverse consequences of interrupted nutrition are well recognized, and the potential benefit of minimizing unnecessary interruptions in nutrition delivery is broad. These consensus recommendations are not proposed without concern for aspiration risk. Accordingly, implementation of these recommendations should be accompanied by appropriate aspiration precautions. Although these recommendations are not intended to guide airway management, the care team should treat patients as being at increased risk for aspiration when clinically appropriate. This includes frequent assessment to ensure that the airway is secure, suctioning of the oropharyngeal cavity as needed, and confirmation that the endotracheal tube or tracheostomy tube is appropriately positioned and the cuff is inflated.

## Conclusions

In this modified Delphi study, through an iterative consensus process, we developed expert clinical practice recommendations to guide periprocedural fasting in patients receiving tube feeds. These recommendations address a gap in existing fasting guidelines for a nutritionally vulnerable population that often experiences repeated interruptions in enteral nutrition for procedures requiring anesthesia or sedation. By balancing aspiration risk with the need to preserve nutrient delivery, these recommendations may help institutions and guide clinicians in supporting safer, more consistent periprocedural nutrition. Future studies should evaluate the impact of the recommended practices and accumulate additional evidence to support the development of future formal guidelines.

## Acknowledgments

The authors respectfully acknowledge the late George Williams (formerly of the UTHealth Houston Department of Anesthesiology, Critical Care, and Pain Medicine, Houston, Texas) for his foundational contributions to this work. A dedicated clinician-scientist, tireless advocate for optimized perioperative nutrition, and valued member of the American Society of Anesthesiologists Committee on Critical Care Medicine, Dr. Williams provided critical guidance and support during the development of the concepts that formed the basis of this consensus effort.

## Research Support

Support was provided solely from institutional and/or departmental sources.

## Competing Interests

Dr. Sparling and Dr. Gupta: Advisory board for Delcath Systems (New York, New York). Dr. Khanna: Medtronic (Minneapolis, Minnesota), Edwards Lifesciences (Irvine, California), Philips Research North America (Cambridge, Massachusetts), Bayer Corporation (Whippany, New Jersey), AOP (Vienna, Austria), GE Healthcare (Chicago, Illinois), Innoviva Therapeutics (Waltham, Massachusetts), Viatris (Canonsburg, Pennsylvania), SERB Pharmaceuticals (West Conshohocken, Pennsylvania), Pharmazz Inc. (Willowbrook, Illinois), ASA Committee on Critical Care Medicine (Committee Chair), SCCM (council member), SCCM Surviving Sepsis Campaign (Research Committee member), SCCM ESICM consensus definition of refractory septic shock (co-chair). Ongoing support Wake Forest CTSI: RAAS dysfunction in septic shock and NIH/NHLBI R01HL177834-01: Dysfunctional Renin Angiotensin System in Septic Shock. Editor: *British Journal of Anaesthesia*. Dr. Wischmeyer: Grants from NIH (R01 HD107103), Department of Defense, Abbott Inc. (Abbott Park, Illinois), Baxter (Deerfield, Illinois), Fresenius Inc. (Bad Homburg, Germany), Protara Inc. (New York, New York) to Duke University. Serving as a consultant for Abbott Inc, Baxter, Danone-Nutricia (Utrecht, Netherlands), Fresenius, ART Medical (Netanya, Israel), Mend Inc. (Long Island City, New York), Marius Pharmaceutical (Raleigh, North Carolina), MuscleSound (Denver, Colorado). Continuing medical education (CME) lectures for Abbott, Baxter, Fresenius, Protara, Danone Nutricia. Unrestricted gift funding for research from MuscleSound. Dr. Yeh: UpToDate (author royalties; Waltham, Massachusetts); Takeda Pharmaceuticals (consultant; Tokyo, Japan); Baxter (consultant); Wave Strategy (consultant; Cleveland, Ohio); Irrimax, LLC (medical advisory board; Lawrenceville, Georgia); Certified Nutrition Support Clinician Exam Committee (chair); National Board of Nutrition Support Certification (Board of Directors); Eli Lilly (Indianapolis, Indiana). Mechanick: Advisory boards of Abbott Nutrition and Twin Health (Menlo Park, California); received honoraria for lectures from Abbott Nutrition and Merck (Rahway, New Jersey). Dr. Nagrebetsky: Principal Investigator of a multicenter clinical trial comparing preoperative fasting practices in enteral nutrition (PCORI BPS-2023C1-31273). Dr. Nasa: Shares in Novo Nordisk (Bagsvaerd, Denmark), Eli Lilly, and AstraZeneca (Cambridge, United Kingdom). Dr. Karamchandani: Philips Healthcare (Cambridge, Massachusetts). Dr. Stoppe: received honorarium as consultant from Fresenius, NUTRICIA, Baxter, B. BRAUN (Melsungen, Germany) and Nestle (Bridgewater, New Jersey) independent from this work; Stoppe received financial support for an IIT independent from this work by Fresenius Kabi. Dr. Rice: NIH NCATS; Nestle Health Sciences. Dr. DeWane: Equity interest in Veradermics (Dermatology BioPharma; New Haven, Connecticut). The other authors declare no competing interests.

## Supplemental Digital Content

Expert panel membership, Supplemental Digital Content1, table 1: https://links.lww.com/ALNO/A29.

Successive results of Delphi rounds, Supplemental Digital Content1, table 2: https://links.lww.com/ALNO/A29.

Recommendations presented to expert panel by round, Supplemental Digital Content1, table 3: https://links.lww.com/ALNO/A29.

## Supplementary Material

## Appendix

Members of the ASA COCCM Task Force on Periprocedural Fasting in Patients Who Receive Tube Feeding: Shahla Siddiqui, MBBS, MSc, Department of Anesthesia, Critical Care, and Pain Medicine, Beth Israel Deaconess Medical Center, Harvard Medical School, Boston, Massachusetts; Roshni Sreedharan, MD, MBA, Integrated Hospital Care Institute, Cleveland Clinic Lerner College of Medicine, Care Western Reserve University, Cleveland, Ohio; Vanessa Moll, MD, PhD, Department of Anesthesiology, University of Minnesota Medical School, Minneapolis, Minnesota; Jovana Rutherford, BSN, Ross University School of Medicine, Bridgetown, Barbados; Amber Campbell, MD, MS, Red Bank Anesthesia, Riverview Medical Center, Red Bank, New Jersey; Lauren Sutherland, MD, Department of Anesthesiology, Columbia University Irving Medical Center, New York, New York; Pingping Song, MD, MS, Department of Anesthesiology and Pain Medicine, University of Washington Medical Center- Montlake, Seattle, Washington; Jayashree Raikhelkar, MD, Department of Anesthesiology, Emory University School of Medicine, Atlanta, Georgia; John Klick, MD, Department of Anesthesiology, University of Vermont Medical Center, Larner College of Medicine, University of Vermont, Burlington, Vermont; Kunal Karamchandani, MD, Department of Anesthesiology and Pain Management, University of Texas Southwest Medical Center, Dallas, Texas; Jarva Chow, MD, MS, MPH, Department of Anesthesia and Critical Care, University of Chicago, Chicago, Illinois; Ashish Khanna, MD, MS, Department of Anesthesiology, Wake Forest University School of Medicine, Winston-Salem, North Carolina; Krassimir Denchev, MD, Department of Anesthesiology, Wayne State University School of Medicine, Detroit, Michigan; Courtney Maxey-Jones, MD, MBA, Department of Medicine, SUNY Upstate Medical University, Syracuse, New York; Joy Lo Chen, MD, Department of Anesthesiology and Pain Management, University of Texas Southwestern Medical Center, Dallas, Texas; Nathan Smischney, MD, MS, Department of Anesthesiology and Perioperative Medicine, Mayo Clinic, Rochester, Minnesota; Jeffrey Carness, MD, Department of Anesthesiology, Madigan Army Medical Center, Tacoma, Washington; Alisha Sachdev, MD, Department of Anesthesiology, Rush University Medical Center, Chicago, Illinois; Mark Nunnally, MD, Department of Anesthesia and Critical Care, University of Chicago, Chicago, Illinois; Ricardo Verdiner, MD, Department of Anesthesiology and Perioperative Medicine, Rutgers Robert Wood Johnscon Medical School, New Brunswick, New Jersey; Graham Lubinsky, MD, Department of Anesthesiology, Advocate Illinois Masonic Medical Center, Chicago, Illinois; Catherine Kuza, MD, Department of Anesthesiology, Harbor-UCLA Medical Center, Torrance, California; R. Alok Gupta, MD, Department of Anesthesioogy, Northwestern University Feinberg School of Medicine, Chicago, Illinois; Proshad Efune, MD, Department of Anesthesiology and Pain Management, University of Texas Southwestern Medical Center, Dallas, Texas; Sam Beiser, BSN, Surgical Intensive Care Unit, Massachusetts General Hospital, Boston, Massachusetts; Alison Rheaume, MS, one99, Franklin, Massachusetts.
