# Supplementary material for: American Society of Anesthesiologists Guidance on Periprocedural Fasting in Patients Receiving Tube Feeding
Source: Anesthesiol Open. 2026 Oct 28;1(3):e0037. doi: 10.1097/ao9.0000000000000037 (PMC13718895; doi:10.1097/ao9.0000000000000037)
Supplement: Supplementary file 1 [file ao9-1-e0037-s001.pdf]

Supplemental Table 1: Expert Panel Membership

Supplemental Table 2: Successive Results of Delphi Rounds

Supplemental Table 3: Recommendations Presented to Expert Panel by Round.

Supplemental Table 1: Expert Panel Membership

Paul Wischmeyer, MD
Duke University, Department of Anesthesiology

Todd Rice, MD, MSc
Vanderbilt University Medical Center, Department of Allergy, Pulmonary and Critical Care Medicine

Alok (Ravindra) Gupta, MD
Department of Anesthesiology at Northwestern University Feinberg School of Medicine

Ronald Pearl, MD, PhD
Stanford University, Department of Anesthesiology, Perioperative and Pain Medicine

Hassan Dashti, PhD
Massachusetts General Hospital, Department of Anesthesia, Critical Care and Pain Medicine

Michael Dewane, MD
Massachusetts General Hospital, Division of Trauma, Emergency Surgery and Surgical Critical Care

Martin Rosenthal, MD
Loma Linda University Health, Department of Surgery

Jeffrey Mechanick, MD
Icahn School of Medicine at Mount Sinai, Department of Medicine

Christian Stoppe, MD
Würzburg University Hospital, Department of Anesthesiology, Intensive Care, Emergency and Pain Medicine

Dante Yeh, MD
University of Colorado, Department of Surgery

Richard Dutton, MD
U.S. Anesthesia Partners

Supplemental Table 2: Successive Results of Delphi Rounds

**Examples: bowel resection, enteroscopy. **Airway instrumentation includes tracheal intubation, tracheal extubation, airway exchange, and other procedures involving the airway.*
****Examples of major motility impairment and structural diseases include gastroparesis, bowel obstruction, ileus, recent administration of glucagon-like peptide-1 receptor agonist (GLP-1RA); compromised lower esophageal sphincter; gastrointestinal bleeding.*
§ No equivalent recommendation from Round 1.
~ Recommendation 5.1 was the only recommendation to undergo a fourth round.

				Round 1			Round 2			Round 3			Round 4		
**Number**	**Patient Population**	**Part**	**Recomendation**	Responses	% disagreement (1-3)	% agreement (5-7)	Responses	% disagreement (1-3)	% agreement (5-7)	Responses	% disagreement (1-3)	% agreement (5-7)	Responses	% disagreement (1-3)	% agreement (5-7)
**1**	**Hospitalized Patients**	1.1	Institutions should implement periprocedural tube feeding protocols based on national recommendations to minimize periprocedural nutrition loss.	11	0%	100%	11	0%	100%	11	0%	100%		~	
		1.2	Institutions should eliminate “NPO after midnight” orders, including default or pre-populated orders.	11	0%	100%	11	9%	82%	11	0%	100%			
**2**			Patients who will be extubated after procedure should follow conventional fasting guidelines (i.e., 6 hours for most tube feeds).	11	9%	64%	11	0%	91%	11	9%	91%			

3	In hospitalized patients WITH a secure airway (cuffed endotracheal tube or cuffed tracheostomy tube) who undergo procedures with sedation or anesthesia that do not require fasting for surgical indications* or involve airway instrumentation:									
3.1	Fasting times for both gastric and post-pyloric feeding tubes should be the same.	11	18%	64%	11	9%	91%	11	0%	100%
3.2	For procedures performed primarily in the supine, head-up (reverse Trendelenburg), lateral, or lithotomy positions, via gastric or post-pyloric tube feeding should continue until departure to the operating room.	§	§	§	11	9%	91%	11	0%	100%
3.3	For patients with a post-pyloric feeding tube having procedures in prone or head-down (Trendelenburg) position, consider	§	§	§	11	9%	73%	11	0%	91%

		continuing tube feeding until departure to the operating room.									
	3.4	Consider uninterrupted periprocedural tube feeding via gastric or post-pyloric feeding tube, including continuing tube feeding during the procedure, especially in patients with high nutritional needs, such as burn patients.	11	27%	73%	11	0%	100%	11	0%	100%
	3.5	Consider suctioning gastric tubes or gastric ports of combined tubes prior to departure to the operating room in procedures with increased risk of regurgitation/vomiting (e.g., abdominal insufflation) or airway loss (e.g., craniofacial procedures).	11	0%	73%	11	0%	91%	11	0%	100%
**4**		In hospitalized patients WITHOUT a secure airway (cuffed endotracheal tube or cuffed tracheostomy tube) who undergo									

	procedures with sedation or anesthesia that do not require fasting for surgical indications* or involve airway instrumentation:**									
4.1	Continue tube feeding until two hours prior to departure to the operating room if a patient has a surgically placed or radiologically confirmed (within 48 hours prior to procedure) post-pyloric feeding tube.	11	0%	82%	11	0%	100%	11	0%	91%
4.2	Suction gastric feeding tube if it permits suctioning prior to departure to the operating room.	11	0%	91%	11	0%	91%	11	0%	91%
4.3	In hospitalized patients who maintain oral intake in addition to tube feeding, clear oral carbohydrate solutions should be encouraged until 2 hours prior to elective procedures.	11	0	91%	11	0%	100%	11	0%	100%
4.4	Consider gastric ultrasonography by a trained provider in cases with uncertain fasting	11	27%	64%	10	10%	70%	11	0%	82%

		time and/or particular concerns for delayed gastric emptying.***									
**5**		In hospitalized patients with documented or diagnosed major impairment of gastrointestinal motility or significant structural disease of the gastrointestinal tract, duration of periprocedural fasting should be determined on a case-by-case basis, following a discussion with the procedural, anesthesiology, and intensive care teams.***	11	0%	100%	10	10%	90%	11	0%	100%
**6**		There are insufficient data to guide periprocedural fasting in hospitalized patients receiving bolus tube feeding, irrespective of the presence of a secure airway. Decisions on the optimal duration of periprocedural fasting in these	11	9	91%	11	9%	82%	11	9%	91%

		patients should be made on a case-by-case basis.									
**7**	7.1	Consider continuing tube feeding until the time of planned extubation in the ICU.	11	9%	82%	11	0%	91%	11	0%	100%
	7.2	Continued tube feeding until the time of planned ICU extubation may be implemented without suctioning/sumping (if Salem Sump tube) the stomach prior to extubation.	11	45%	36%	11	18%	82%	11	0%	91%
**8**		Patients with a secure airway undergoing lower GI tract procedures (e.g., endoscopy, bowel surgery) may continue to receive enteral nutrition until the time of procedure if approved by the procedural team.	11	9%	64%	11	9%	73%	11	0%	91%
**9**		Patients with a secure airway undergoing tracheostomy may continue to receive tube feeding until the time of procedure if approved by the procedural and	11	18%	64%	11	9%	73%	11	0%	91%

			anesthesiology teams.												
**10**	**Non-hospitalized Patients**		In non-hospitalized patients who undergo procedures with sedation or anesthesia	11	9%	82%									
		10.1	Tube feeding should be held 6 hours prior to onset of sedation or anesthesia.	§	§	§	11	27%	55%	11	0%	100%	11	0	100%
		10.2	Fasting for food and liquids should follow existing periprocedural fasting guidelines.	§	§	§	11	0%	91%	11	9%	91%		~	

Supplemental Table 3: Recommendations Presented to Expert Panel by Round

Note: No changes to statements between rounds 3 and 4. Note table continues with changes post steering committee and task force feedback below.

Round 1			Round 2			Round 3
**Number**	Part	Recommendation	Number	Part	Recommendation	Recommendation
**1**		Institutions should develop and implement preprocedural and periprocedural tube feeding protocols to establish uniformly acceptable practices.	1	1.1	Institutions should implement periprocedural tube feeding protocols based on national recommendations to minimize periprocedural nutrition loss.	Institutions should implement periprocedural tube feeding protocols based on national recommendations to minimize periprocedural nutrition loss.
**2**		Minimize “NPO after midnight” orders, including default or pre-populated orders, to avoid unnecessary interruptions in nutrient delivery.		1.2	Institutions should eliminate “NPO after midnight” orders, including default or pre-populated orders.	Institutions should eliminate “NPO after midnight” orders, including default or pre-populated orders.
**3**		Patients with a secure airway should have the same fasting times for gastric vs post-pyloric tubes.	2		In hospitalized patients with a secure airway (cuffed endotracheal tube or cuffed tracheostomy tube) who undergo procedures with sedation or anesthesia that do not require fasting for surgical indications* or airway removal:<br>*Examples: bowel resection, endoscopy	In hospitalized patients with a secure airway (cuffed endotracheal tube or cuffed tracheostomy tube) who undergo procedures with sedation or anesthesia that do not require fasting for surgical indications* or airway removal:<br>*Examples: bowel resection, endoscopy
**4**		In hospitalized patients with a secure airway (cuffed endotracheal tube or cuffed tracheostomy tube) who undergo non-gastrointestinal tract, non-lung, non-airway procedures in the supine position for most of the procedure duration, tube feeding should continue until departure to the operating room.		2.1	Fasting times for gastric and post-pyloric feeding tubes should be the same.	Fasting times for gastric and post-pyloric feeding tubes should be the same.
	a	Consider for procedures in the *reverse Trendelenburg (head up) position*…		2.2	Tube feeding should continue until departure to the operating	Tube feeding should continue until departure to the operating room for

				room for procedures performed primarily in the supine, head-up, lateral, or lithotomy positions.	procedures performed primarily in the supine, head-up, lateral, or lithotomy positions.
b	Consider for procedures in the *Trendelenburg (head down) position…*		2.3	For procedures in prone or head-down (Trendelenburg) position, consider continuing tube feeding until departure to the operating room in patients with a post-pyloric feeding tube.	For procedures in prone or head-down (Trendelenburg) position, consider continuing tube feeding until departure to the operating room in patients with a post-pyloric feeding tube.
c	Consider for procedures the *in prone position…*		2.4	Consider uninterrupted perioperative tube feeding via gastric or post-pyloric feeding tube, including tube feeding during the procedure, especially in patients with high nutritional needs such as burn patients.	Consider uninterrupted perioperative tube feeding via gastric or post-pyloric feeding tube, including tube feeding during the procedure, especially in patients with high nutritional needs such as burn patients.
d	Consider for procedures in the *lateral position…*		2.5	Consider suctioning gastric tubes or gastric ports of combined tubes prior to departure to the operating room in cases with increased risk of regurgitation/vomiting (e.g., abdominal insufflation) or airway loss (e.g., craniofacial procedures).	Consider suctioning gastric tubes or gastric ports of combined tubes prior to departure to the operating room in cases with increased risk of regurgitation/vomiting (e.g., abdominal insufflation) or airway loss (e.g., craniofacial procedures).
e	Consider for procedures in the *lithotomy position…*	3		In hospitalized patients WITHOUT a secure airway (cuffed endotracheal tube or cuffed tracheostomy tube) who undergo procedures with sedation or anesthesia that do not require fasting for surgical indications* or airway removal:	In hospitalized patients WITHOUT a secure airway (cuffed endotracheal tube or cuffed tracheostomy tube) who undergo procedures with sedation or anesthesia that do not require fasting for surgical indications* or airway removal:

				*Examples: bowel resection, enteroscopy	*Examples: bowel resection, enteroscopy
**5**		Patients undergoing procedure in positions other than supine and level should follow conventional 8-hour fasting periods for tube feeding, solid food, and non-clear liquids. *(Note to experts: this recommendation contradicts recommendations 4a-d. These contradicting options are included to assess support for either option.)*	3.1	Suction gastric feeding tube if it permits suctioning prior to departure to the operating room.	Suction gastric feeding tube if it permits suctioning prior to departure to the operating room.
**6**		Clinical teams should consider uninterrupted perioperative tube feeding via post-pyloric tubes in hospitalized with a secure airway, including tube feeding during the procedure, especially in hospitalized with high nutritional needs such as burn patients.	3.2	Continue tube feeding until two hours prior to departure to the operating room if a patient has a surgically placed or radiologically confirmed (within 48 hours prior to procedure) post-pyloric feeding tube	Continue tube feeding until two hours prior to departure to the operating room if a patient has a surgically placed or radiologically confirmed (within 48 hours prior to procedure) post-pyloric feeding tube
	a	Clinical teams should consider uninterrupted perioperative tube feeding via gastric (pre-pyloric) tubes in hospitalized with a secure airway, including tube feeding during the procedure, especially in hospitalized with high nutritional needs such as burn patients.	3.3	In hospitalized who maintain oral intake in addition to tube feeding, clear oral carbohydrate solutions should be encouraged until 2 hours prior to elective procedures.	In hospitalized who maintain oral intake in addition to tube feeding, clear oral carbohydrate solutions should be encouraged until 2 hours prior to elective procedures.
**7**		In hospitalized patients with a secure airway, if feeding tube permits suctioning, suction gastric (pre-pyloric) tubes prior to transferring to the operating room (unless planning for uninterrupted perioperative tube feeding).	3.4	Consider gastric ultrasonography by a trained provider in cases with uncertain fasting time and/or particular concerns for gastric emptying.	Consider gastric ultrasonography by a trained provider in cases with uncertain fasting time and/or particular concerns for gastric emptying.

	a	If feeding tube permits suctioning, suction *post-pyloric* tubes...	4		In hospitalized patients with documented or diagnosed major impairment of gastrointestinal motility or significant structural disease of the gastrointestinal tract, duration of preprocedural fasting should be determined on a case-by-case basis, following a discussion with the procedural ,anesthesiology, and intensive care teams. (Examples of major motility impairment and structural diseases are presented in Caveats to Consider of the current recommendations)	In hospitalized patients with documented or diagnosed major impairment of gastrointestinal motility or significant structural disease of the gastrointestinal tract, duration of preprocedural fasting should be determined on a case-by-case basis, following a discussion with the procedural, anesthesiology, and intensive care teams. (Examples of major motility impairment and structural diseases are presented in Caveats to Consider of the current recommendations)
	b	If, in addition to a post-pyloric feeding tube, a patient has a *gastric tube or a gastric port of a combined tube*, clinicians should consider suctioning gastric contents...	5		In non-hospitalized patients who undergo procedures with sedation or anesthesia	In non-hospitalized patients who undergo procedures with sedation or anesthesia
	d	In hospitalized *without a secure airway*, if feeding tube permits suctioning, suction gastric tubes...		5.1	Tube feeding should be held 8 hours prior to onset of sedation or anesthesia	Tube feeding should be held 6 hours prior to onset of sedation or anesthesia
	d	In hospitalized *without a secure airway*, if feeding tube permits suctioning, suction *post-pyloric* tubes...		5.2	Fasting for food and liquids should follow existing preoperative fasting guidelines	Fasting for food and liquids should follow existing preoperative fasting guidelines
**8**		In hospitalized patients without a secure airway who have a surgically placed or radiologically confirmed (within 48 hours prior to procedure) post-pyloric feeding tube, tube feeding should be continued until two hours	6		There is insufficient data to guide preoperative fasting in hospitalized patients receiving bolus tube feeding irrespective of the presence of a secure airway. Decisions on the optimal duration of	There is insufficient data to guide preoperative fasting in hospitalized patients receiving bolus tube feeding irrespective of the presence of a secure airway. Decisions on the optimal duration of preoperative

	prior to transferring to the operating room.		preoperative fasting in these patients should be made on a case-by-case basis.	fasting in these patients should be made on a case-by-case basis.
**9**	In hospitalized without a secure airway, consider ultrasonographic assessment of gastric volume in cases with uncertain fasting time and/or particular concerns for gastric emptying, if adequate expertise is available.	7	Patients who will be extubated after procedure should follow conventional fasting guidelines (i.e., 6 hours for most tube feeds).	Patients who will be extubated after procedure should follow conventional fasting guidelines (i.e., 6 hours for most tube feeds).
**10**	In hospitalized with documented or diagnosed major impairment of gastrointestinal motility or significant structural disease of the gastrointestinal tract, duration of preprocedural fasting should be determined on a case-by-case basis, following a discussion with the procedural and anesthesiology/intensive care teams. (Examples of major motility impairment and structural diseases are presented in Caveats to Consider of the current recommendations)	8	Consider continuing tube feeding until the time of planned extubation in the ICU.	Consider continuing tube feeding until the time of planned extubation in the ICU.
**11**	Non-hospitalized patients should follow conventional 8-hour fasting periods for tube feeding since the risk of holding tube feeding is likely lower in non-hospitalized patients receiving tube feeding. Fasting periods for solid food and clear liquids should follow existing guidelines.	9	Continued tube feeding until the time of planned ICU extubation may be implemented without suctioning/sumping the stomach prior to extubation.	Continued tube feeding until the time of planned ICU extubation may be implemented without suctioning/sumping the stomach prior to extubation.
**12**	Data are lacking to guide preoperative fasting in hospitalized patients receiving bolus tube feeding irrespective of the presence of a secure airway. Decisions on the optimal	10	Patients with a secure airway undergoing lower GI tract procedures (e.g., endoscopy, bowel surgery) may continue to receive enteral nutrition until	Patients with a secure airway undergoing lower GI tract procedures (e.g., endoscopy, bowel surgery) may continue to receive enteral nutrition

	duration of preoperative fasting in these patients should be made on a case-by-case basis.		the time of procedure if approved by the procedural team.	until the time of procedure if approved by the procedural team.
**13**	Patients who will be extubated after procedure should follow conventional fasting guidelines (i.e., 8 hours for most tube feeds).	11	Patients with a secure airway undergoing tracheostomy may continue to receive tube feeding until the time of procedure if approved by the procedural and anesthesiology teams.	Patients with a secure airway undergoing tracheostomy may continue to receive tube feeding until the time of procedure if approved by the procedural and anesthesiology teams.
**14**	Clinicians should consider continuing tube feeding until the time of planned extubation in the ICU.			
**15**	Continued tube feeding until the time of planned ICU extubation may be implemented without suctioning/sumping the stomach prior to extubation.			
**16**	Patients with a secure airway undergoing lower GI tract procedures may continue to receive enteral nutrition until the time of procedure if approved by the procedural team.			
**17**	In hospitalized who maintain oral intake in addition to tube feeding, clear oral carbohydrate solutions should be encouraged until 2 hours prior to elective procedures.			
**18**	Patients undergoing tracheostomy may continue to receive tube feeding until the time of procedure if approved by the procedural and anesthesiology teams.			
